# Analysis of a Sabin-Strain Inactivated Poliovirus Vaccine Response to a Circulating Type 2 Vaccine-Derived Poliovirus Event in Sichuan Province, China 2019-2021

**DOI:** 10.1001/jamanetworkopen.2022.49710

**Published:** 2023-01-05

**Authors:** Hong Yang, Qi Qi, Yong Zhang, Ning Wen, Lei Cao, Yu Liu, Chunxiang Fan, Dongmei Yan, Xiaoping Zhu, Lixin Hao, Shuangli Zhu, Qianli Ma, Jiajie Liu, Chao Ma, Lei Nan, Yong Chen, Xiaozhen Ma, Na Chen, Kun Deng, Ge Shao, Xianxiang Ding, Zhijie An, Lance E. Rodewald, Xiaolei Li, Dongyan Wang, Hui Zhu, Huaqing Wang, Zijian Feng, Wenbo Xu, Jiushun Zhou, Zundong Yin

**Affiliations:** 1 National Immunization Program, Chinese Center for Disease Control and Prevention, Beijing, China; 2 Sichuan Provincial Center for Disease Control and Prevention, Chengdu, China; 3 Institute for Viral Disease Control and Prevention, Chinese Center for Disease Control and Prevention, Beijing, China; 4 Liangshan Prefectural Center for Disease Control and Prevention, Liangshan, China; 5 Leibo County Center for Disease Control and Prevention, Liangshan, China; 6 Chinese Field Epidemiology Training Program, Beijing, China; 7 Chinese Center for Disease Control and Prevention, Beijing, China

## Abstract

**Importance:**

The Sabin-strain inactivated poliovirus vaccine (IPV) may be a tool for polio outbreak response in certain situations.

**Objective:**

To investigate the response to a type 2 vaccine-derived poliovirus (VDPV2) outbreak.

**Design, Setting, and Participants:**

This case series was conducted in China after a VDPV2 was detected in stool specimens from a child with acute flaccid paralysis (AFP) in Sichuan Province in 2019, 3 years after the global withdrawal of live, attenuated type 2 oral poliovirus vaccine (OPV). Investigation followed National Health Commission and World Health Organization guidance and included searching hospitals for unreported AFP cases; testing stool specimens from the child, his contacts, and local children; enhanced environmental surveillance for VDPV2s in wastewater; and measuring vaccination coverage. Sabin-strain IPV campaigns were conducted in a wide geographic area.

**Main Outcomes and Measures:**

Any VDPV2 detection after completion of the supplementary immunization activities.

**Results:**

A 28-nucleotide-change VDPV2 was isolated from a young boy. Three VDPV2s were detected in healthy children; 2 were contacts of the original child, and none had paralysis. A search of 31 million hospital records found 10 unreported AFP cases; none were polio. No type 2 polioviruses were found in wastewater. Prior to the event, polio vaccine coverage was 65% among children younger than 5 years. Sabin-strain IPV campaigns reached more than 97% of targeted children, administering 1.4 million doses. No transmission source was identified. More than 1 year of enhanced poliovirus environmental and AFP surveillance detected no additional VDPVs.

**Conclusions and Relevance:**

These findings suggest that the circulating VPDV2 outbreak in 2019 was associated with low vaccine coverage. An investigation discovered 3 infected but otherwise healthy children and no evidence of the virus in wastewater. Following Sabin-strain IPV–only campaigns expanding from county to prefecture, the poliovirus was not detected, and the outbreak response was considered by an expert panel and the World Health Organization to have been successful. This success suggests that the Sabin-strain IPV may be a useful tool for responding to circulating VDPV2 outbreaks when high-quality supplementary immunization activities can be conducted and carefully monitored in settings with good sanitation.

## Key Points

**Question:**

Can the Sabin-strain of the inactivated poliovirus vaccine (IPV) be used in response to a circulating type 2 vaccine-derived poliovirus (VDPV2) event?

**Findings:**

In this case report of a circulating VDPV2 outbreak that occurred in Sichuan, China, in 2019, investigation found that the outbreak was enabled by low population immunity against type 2 polio due to low, routine 1-dose coverage with IPV. Environmental and acute flaccid paralysis surveillance activities were heightened for more than 1 year after 2 rounds of supplementary immunization activities, and the VDPV2 was not detected.

**Meaning:**

These findings suggest that the Sabin-strain IPV can be used in response to VDPV2 outbreaks.

## Teaser

This case series investigates the investigation of, response to, and surveillance following a type 2 vaccine-derived poliovirus (VDPV2) outbreak.

## Introduction

Since 2014, the World Health Organization (WHO) has continuously declared polio to be a public health emergency of international concern. The Global Polio Eradication Initiative (GPEI) has been making good progress toward worldwide eradication of polio. Wild poliovirus (WPV) types 2 and 3 were declared eradicated in 2015 and 2019, and 5 of the 6 WHO regions have been declared polio free. Most countries used Sabin-strain live attenuated oral poliovirus vaccine (OPV) for routine immunization, but Sabin-strain viruses, on rare occasions, revert to transmitting neurovirulent forms during replication in vaccinees and contacts, making it impossible to eradicate polio with the current OPVs alone.

Low population immunity enables generation and transmission of vaccine-derived polioviruses (VDPVs). One year after WPV type 2 was declared eradicated, type 2 OPV was removed from trivalent live attenuated OPV in all OPV-using countries to avoid generating type 2 VDPVs that circulate and cause outbreaks (circulating VDPV2s). Bivalent OPVs were substituted for trivalent OPVs, and trivalent inactivated poliovirus vaccine (IPVs) were introduced in OPV-using countries. Protection from type 2 polioviruses could then only come from an IPV, initially recommended as a single dose in most bivalent OPV-using countries, including China, compared with 3 to 5 doses in countries using only IPV for routine immunization.

Infectious diseases modelers estimated that a small number of VDPV2 outbreaks would occur in the months following type 2 OPV cessation, mainly from undetected circulating VDPV2s generated by trivalent OPV use prior to type 2 OPV withdrawal.^1^ These circulating VDPV2 outbreaks were to be responded to with monovalent type 2 OPVs on the recommendation of the WHO director general and release from the WHO monovalent type 2 OPV stockpile. However, monovalent type 2 OPV is a live virus vaccine, the same Sabin-strain type 2 OPV that was in trivalent OPV, and therefore can seed additional VDPV2 and cause circulating VDPV2 outbreaks, threatening global polio eradication. Since conditions favoring generation of VDPV2s (low population immunity) are generally present in outbreak settings, use of a monovalent type 2 OPV to respond to a type 2 polio outbreak carries substantial risk. Indeed, the number of type 2 polio outbreaks following the cessation of type 2 OPV far exceeded projections by the modelers due to seeding of new outbreaks from monovalent type 2 OPV use. Although IPV cannot seed new polioviruses, it is not recommended by the WHO for responding to type 2 circulating VDPV outbreaks because it does not block infection and transmission as effectively as OPV. A recently published scientific review by Estivarez and colleagues^2^ on the use of IPV in campaigns to control circulating VDPV2 outbreaks said that addition of IPV to type 2 OPV campaigns will slow the implementation of the response, will have a small impact on reduction of paralytic cases at a high programmatic cost, and will not close immunity gaps persisting due to poor quality of vaccination activities.^2^

On June 16, 2019, 3 years after global type 2 OPV cessation, a VDPV2 was detected in stool specimens from a child in China who had acute flaccid paralysis (AFP). Any detection of type 2 poliovirus is a major concern, signaling a VDPV2 event and demanding thorough investigation and response. Local and national Centers for Disease Control and Prevention (CDCs) investigated the outbreak and conducted emergency vaccination, with WHO concurrence, with the then-recently licensed trivalent Sabin-strain IPV (IPV) rather than monovalent type 2 OPV. We report the results of the investigation, response, and follow-up surveillance.

## Methods

### Ethics

In China, polio is a level 2 infectious disease. It is the responsibility of disease prevention and control institutions to investigate and respond to any polio event in China. Individual-level, case-based AFP surveillance is mandatory. Polio investigations and responses, such as this case series, are therefore exempted from ethical review and the requirement for informed consent.

### Investigation

The investigation was based on guidelines from China’s National Health Commission, consistent with WHO polio outbreak guidelines. The guidelines require reporting wild and vaccine-derived poliovirus detections to WHO, investigating the clinical circumstances and vaccination histories of AFP cases, conducting active AFP surveillance in hospitals, searching for poliovirus in healthy children and wastewater, assessing polio vaccination coverage levels, reviewing accrued evidence with a panel of polio experts, and using expert panel recommendations to guide response measures and longer-term surveillance for polioviruses.

### Setting

The setting was mainland China, which has been certified by WHO as polio free since 2000, when the entire Western Pacific Region was certified polio free. The AFP case was identified in Leibo County of Liangshan Prefecture of Sichuan Province, located in southwest China. Leibo County is a remote, rural, impoverished area in the southeast of Sichuan that borders Yunnan Province. The population of Liangshan is 5.3 million, 53% of whom are Yi minority individuals. Liangshan is surrounded by 6 prefectures: Ya’an and Ganzi on the north; Leshan, Yibin, and Zhaotong (of Yunnan Province) on the east; and Panzhihua on the south.

Prior to type 2 OPV cessation, China’s routine polio vaccination schedule was 4 doses of trivalent OPV, given at the ages of 2, 3, and 4 months and 4 years. Starting May 2016 and through the study period, the polio vaccination schedule was 1 dose of IPV at age 2 months followed by 3 doses of bivalent (types 1 and 3) OPV at ages 3 and 4 months and 4 years.^3^ In January 2020, based in part on this outbreak, the polio schedule was changed to IPV at ages 2 and 3 months followed by bivalent (types 1 and 3) OPV at ages 4 months and 4 years.

Detection of AFP cases relies on passive reporting from hospitals and active surveillance by county CDC staff. Nearly all hospitals at the county level and greater report to the AFP surveillance system. County CDC staff investigate AFP cases, collect stool specimens, and transport specimens to provincial laboratories for virus isolation. Positive isolates are sent to the National Poliovirus Laboratory at the Chinese CDC, a WHO polio regional reference laboratory, for intratypic differentiation and genome sequencing.

### Sample Testing

Stool samples were tested using WHO-recommended standard practices. Specimens were sent to the WHO-accredited Sichuan provincial polio laboratory for primary screening. Stool samples from the AFP case were cultured in L20B, Rhabdomyosarcoma (RD), and HEp-2 cells. Stool samples from healthy children were tested with real-time polymerase chain reaction (RT-PCR) by Sichuan provincial laboratory. Samples with positive cultures or that have positive RT-PCR results for polioviruses are further characterized by genomic sequencing at the National Polio Reference Laboratory in China CDC, which is a designated WHO Regional Reference Laboratory.

## Results

### Investigation and Response Measures

The index case was a young boy with acute paralysis who was hospitalized for encephalitis on April 2019, thus bringing him to the attention of Liangshan AFP surveillance. He was born in 2014 and lived in Tianba village, Yuanbaoshan Township, Leibo County, Liangshan Prefecture. The child lived in a single-family house in a 2-story building with an outdoor flush toilet that is discharged directly to a field. He received trivalent OPV at ages 4 and 6 months but received no other poliovirus vaccines. Stool samples obtained after hospital discharge were tested by Sichuan CDC and found to contain type 2 Sabin-like poliovirus on June 10 to 11, 2019.

**Figure 1.**
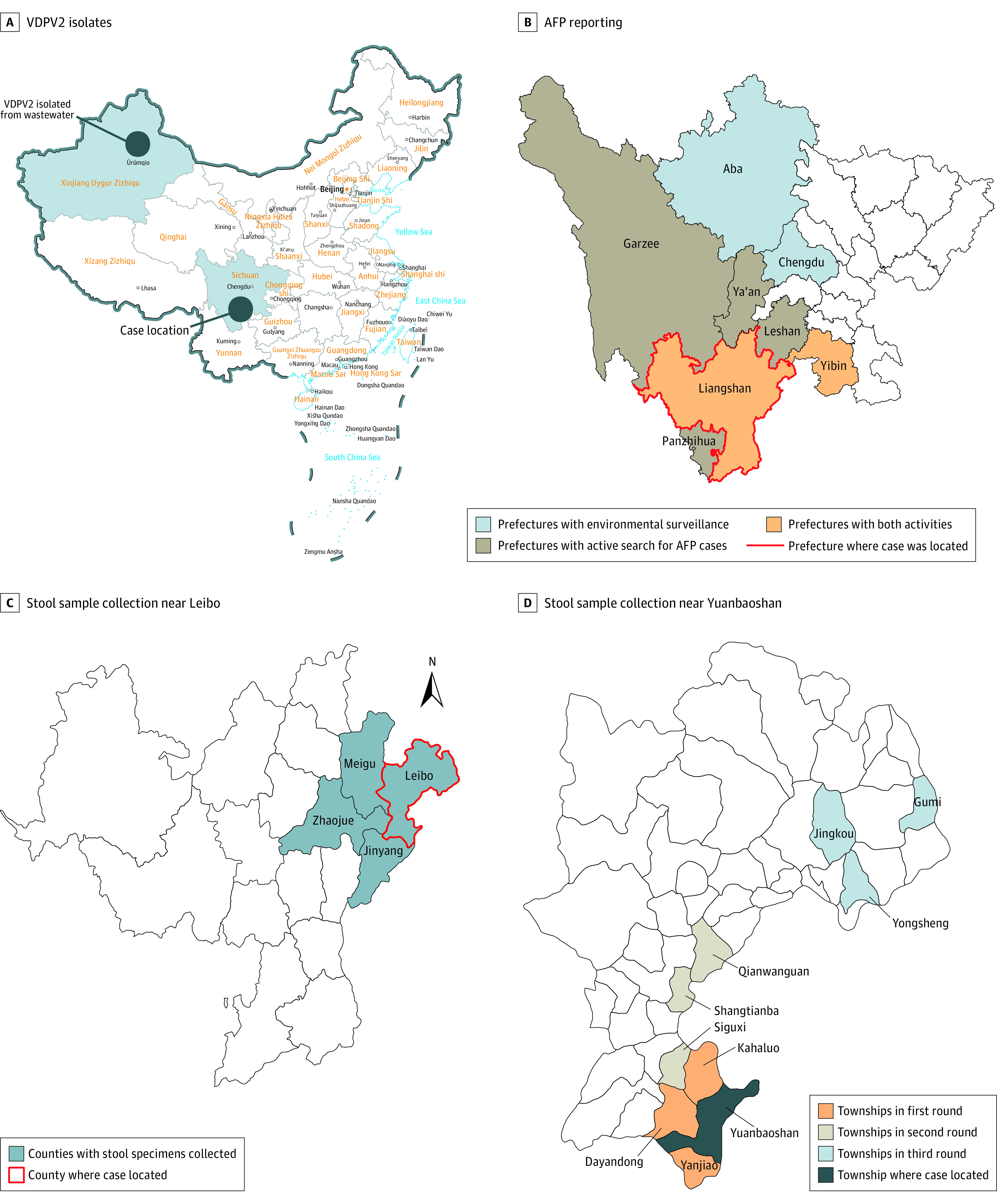
Geographic Reach of the Cases and Surveillance AFP indicates acute flaccid paralysis; VDPV2, type 2 vaccine-derived poliovirus.

**Figure 2.**
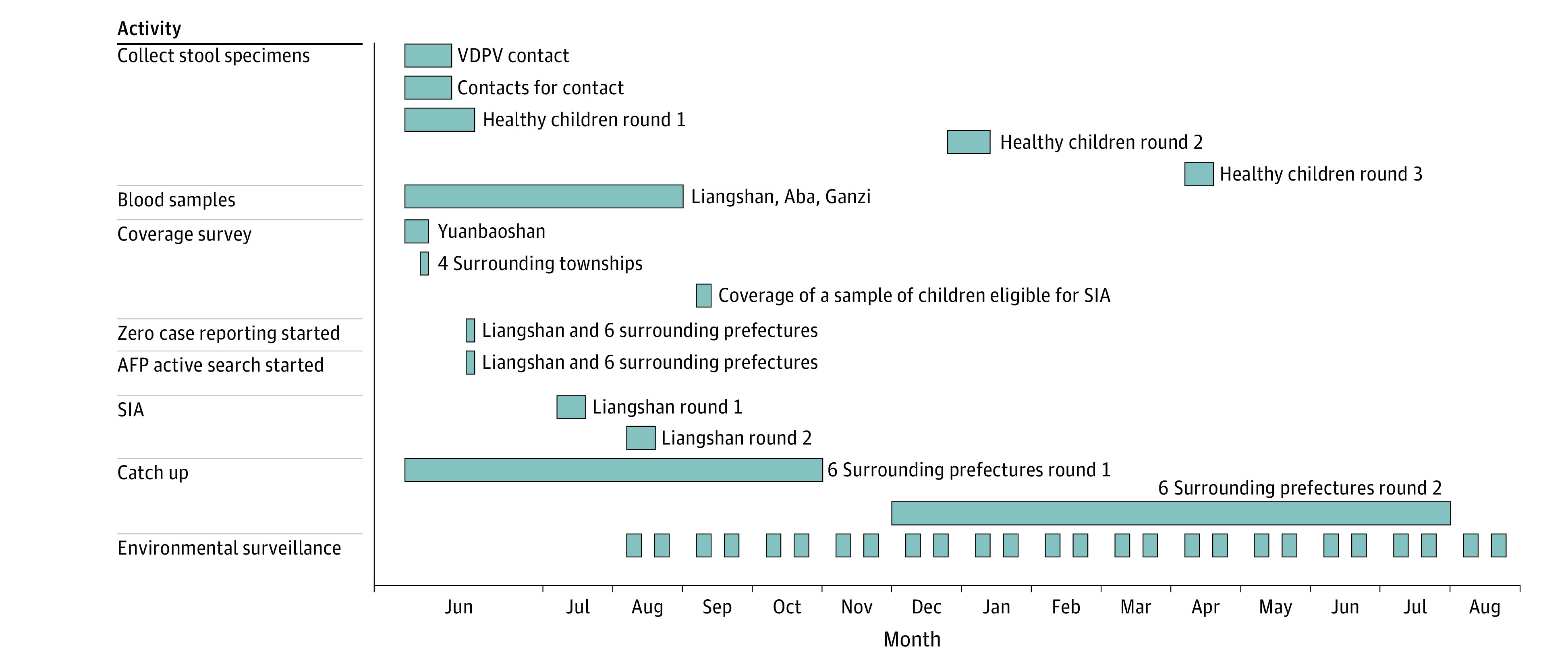
Timeline of Response AFP indicates acute flaccid paralysis; SIA, supplementary immunization activity; VDPV, vaccine-derived poliovirus.

The National Poliovirus Laboratory determined the isolate was a VDPV2, with 28 VP1-region nucleotide changes from the Sabin type 2 vaccine virus. Sequence analysis showed that this VDPV2 had 9 nucleotide changes in common with a VDPV2 isolated from wastewater in Urumqi, Xinjiang Province, in April 2018, which had 13 nucleotide changes from the Sabin type 2 vaccine strain (Figure 1A). The 2 VDPV2s were therefore derived from a common ancestral virus. Since these discoveries were separated in time and space, the VDPV2 had been circulating and was classified as a circulating VDPV2, the presence of which is a major public health emergency. The Chinese CDC designated a level 2 public health emergency response, the second highest response level in China, on June 20, 2019. The timeline of the response is depicted in Figure 2.

### Strengthening AFP Surveillance

Zero-case AFP reporting was started on June 21, 2019, in Liangshan Prefecture and 6 surrounding prefectures, requiring daily or weekly reports of the number of AFP cases discovered, even if zero. Yibin Prefecture, Panzhihua Prefecture, Ya’an Prefecture, Leshan Prefecture, and Ganzi Prefecture started zero-case weekly reporting on June 21, and Aba Prefecture started weekly reporting on August 19 (Figure 1B).

Liangshan and 5 surrounding prefectures conducted medical records–based active search for AFP cases in county-level and greater general hospitals, township health centers, and private hospitals from June 21 to 27, 2019 (Figure 1B). The search targeted individuals with AFP after May 1, 2016; medical record searches included inpatient and outpatient records. Records were text-searched for symptoms such as paralysis, muscle weakness, and limp. Overall, 778 medical institutions were searched, and 31 631 487 medical records were checked. The search identified 289 individuals with AFP, of whom 279 had been reported to the AFP surveillance system. Ten had not been reported: 5 in Liangshan Prefecture and 5 in Yibin Prefecture. Expert review determined that none of the individuals with nonreported AFP had polio (Table).

**Table.** Expert Group Diagnostic Classification of the 10 Unreported AFP Cases

Patient No.	Birth year	Paralysis date	History of polio vaccination	Residual paralysis	Clinical expert diagnosis	Expert group determination	Remarks
1	2005	Jun 28, 2018	2 Doses	Yes	Myelitis	Polio excluded	NA
2	2016	Sep 18, 2017	3 Doses	No	Synovitis	Polio excluded	AFP excluded
3	2017	Feb 1, 2019	Unknown	No	Myositis	Polio excluded	NA
4	2004	Apr 27, 2016	Unknown	No	Periodic paralysis	Polio excluded	NA
5	2016	Aug 7, 2017	3 Doses	Yes	Cerebral palsy	Polio excluded	AFP excluded
6	2013	May 28, 2018	4 Doses	No	Inherited metabolic disease	Polio excluded	AFP excluded
7	2014	Sep 18, 2016	4 Doses	No	Synovitis	Polio excluded	AFP excluded
8	2017	Jun 5, 2018	3 Doses	No	Inherited metabolic disease	Polio excluded	AFP excluded
9	2003	Nov 8, 2017	Unknown	No	Acute myelitis	Polio excluded	NA
10	2012	Sep 8, 2016	Unknown	No	Guillain-Barré syndrome	Polio excluded	NA

Abbreviations: AFP, acute flaccid paralysis; NA, not applicable.

Liangshan and 5 surrounding prefectures conducted community searches for AFP cases, making inquiries of 2 094 404 household members in 10 485 communities. There are 47 townships in the county where the VDPV case was identified. AFP search in the community of the VDPV case’s township and 4 surrounding townships was conducted in combination with a vaccine coverage survey during visits to families. During the search, residents seen on the street were asked whether there were any children with paralysis or a limp or whether there were any children who were unable to walk. In the other 42 townships, community AFP search was conducted through asking residents during walks in the township whether there were any local children with paralysis. Through these searches, 17 children were found to have paralysis; 16 were previously reported to the AFP system and 1, from Liangshan, had not been reported.

### Virus Surveillance in Healthy Children and Wastewater

Laboratory isolation of poliovirus in stool specimens and wastewater was used to search for individual-, community-, and population-level evidence of poliovirus. Stool specimens were obtained from the index child, his contacts, patients where he had been hospitalized (132 children; June 13-18), and healthy children in nearby communities (28 children; June 27-28). Subsequent stool collections were based on laboratory test results from the most recent stool specimens, following up positive isolates in wastewater and children. During August 13-21, 300 stool specimens were obtained from 50 healthy children younger than 5 years—10 specimens for each age cohort of healthy children from counties that neighbor Leibo (Figure 1C) and townships that neighbor Yuanbaoshan (Figure 1D).

Three stool specimens were positive for the sought-for VDPV2. One child was a close contact of the index case, and 1 was a close contact of this close contact. The third child was from Yanjiao Township and was not a contact. All follow-up stool samples from the 3 healthy children were negative, including 4 consecutive samples obtained at least 1 month after the first sample from the index case. No additional stool sample were obtained.

After detection of VDPV from index case, we started the first round of environmental surveillance during August 2019 to January 2020 in 4 sites (Figure 1B), including Liangshan Prefecture (the index case’s residence), Aba (Ngawa) Tibetan and Qiang Autonomous Prefecture (which had a circulating VDPV2 in 2011-2012), Yibin Prefecture (bordering Liangshan and where the index case was seen by a doctor), and Chengdu Prefecture (the capital of Sichuan). Two wastewater samples were collected from each location monthly for 6 consecutive months. In total, 48 wastewater samples were collected, and no VDPVs were detected. This evidence was accepted by polio experts at WHO headquarters and the Western Pacific regional office.

### Polio Vaccine Coverage

Vaccination coverage assessments were conducted among children aged 1 to 5 years through house-to-house surveys June 13 to 15, 2019, in the Tianba village of Yuanbaoshan Township (88 children, all children aged 1-5 years in the township), the home of the index case, and 4 surrounding townships (164 children, at least 30 children in each township). Children’s official vaccination certificates were checked for evidence of polio vaccination. In Tianba, 47 children (53.4%) had received zero doses, 31 (35.2%) received 1 dose, 6 (6.88%) received 2 doses, 3 (3.4%) received 3 doses, and 1 (1.1%) received 4 doses. In the surrounding townships a total of 164 children under 6 years were surveyed, and 106 (65.0%) received 3 doses and 17 of 39 eligible for the fourth dose (43.6%) received 4 doses. In the surrounding township survey, we were unable to determine type 2–specific coverage due to incomplete records, some of which did not distinguish bivalent OPV from IPV.

### Expert Review of Evidence and Recommended Response Measures

On July 4, 2019, a panel of experts in epidemiology, immunization, emergency response, laboratory, polio, and clinical pediatric neurology was assembled as a national experts committee. This panel reviewed the evidence from the investigation and concluded that the virus was likely to continue to spread if unchecked. In addition to standard WHO recommendations for a type 2 poliovirus event, the experts recommended expanding wastewater surveillance, conducting a serological survey to augment coverage assessments,^4^ and conducting outbreak response immunization supplementary immunization activities (SIAs) with Sabin-strain IPV rather than monovalent type 2 OPV.

### Emergency Vaccination

To ensure that all children received at least 2 doses of Sabin-strain IPV, Liangshan CDC conducted 2 SIAs among children aged 2 months to five years (ie, children born between July 1, 2013, and April 30, 2019), excluding children with documentation of 4 doses of IPV or trivalent OPV. Children without contraindications, regardless of their birthplace or registration place, received at least 2 doses of IPV. The SIAs were separated by 1 month, with the first round from July 2 to 14, 2019, and the second round from August 8 to 18, 2019, with a goal of achieving 95% coverage of the target populations at the county level for each SIA. First-round target population coverage was 97.4% (476 815 children vaccinated); second round target population coverage was 98.8% (488 803 children vaccinated).

Between June and October 2019, the 6 prefectures surrounding Liangshan conducted catch-up vaccination with Sabin-strain IPV. Children aged 2 months to 5 years with no history of IPV receipt after the switch from trivalent to bivalent OPV (34 518 children) received 1 dose of IPV; the resulting target group coverage was 97.1%. The surrounding prefectures conducted a second Sabin-strain IPV SIA between December 2019 and July 2020 among children aged 2 months 5 five years (409 280 children; target coverage, 98.6%) to ensure that children in the population had at least 2 doses of poliovirus vaccine containing type 2 polio vaccine.

In September 2019, after the SIAs were completed, coverage was assessed in a sample of SIA-eligible children. Two counties were selected at random from Liangshan Prefecture; 5 townships were randomly selected from the 2 selected counties; 5 administrative villages were randomly selected from each township (if the township had fewer than 5 villages, all were selected); and at least 18 target children were selected from each village, for a total of 943 children. This assessment found 1 or more dose coverage to be 99.9% and 2 or more dose coverage to be 99.2%; coverage in the 2 selected counties and townships were greater than 95.0% at the township level.

### Post-SIA Monitoring

From July to December 2019, the reported AFP incidence was 2.47 per 100 000 in Sichuan Province and 2.23 per 100 000 in Liangshan Prefecture. From January to June 2020 (the first 6 months of COVID-19), the reported AFP incidence was 1.38 per 100 000 in Sichuan and 1.13 per 100 000 in Liangshan. During the first 12 months after the detection of the outbreak (July 2019 to June 2020), reported AFP incidences were 1.92 per 100 000 in Sichuan and 1.67 per 100 000 in Liangshan, exceeding sensitivity requirements of the WHO and China’s National Health Commission.

In 2019, there were 17 AFP cases for which qualified stool specimens were not obtained, and from January to April 2020, there were 2 AFP cases without qualified stool specimens. The expert diagnostic team determined that 18 of these AFP cases were not poliomyelitis; the 19th was the index case of the outbreak.

As of November 12, 2020, in 420 daily AFP reports from Liangshan Prefecture, 27 AFP cases were reported. In 300 weekly reports from the surrounding prefectures, a total of 97 AFP cases were reported from Yibin, Panzhihua, Ya’an, Leshan, and Ganzi prefectures. From 52 weekly AFP reports from Aba Prefecture, 4 AFP cases were reported, and none were polio.

Between December 25, 2019, and January 9, 2020, a second round of community virus surveillance was conducted in the counties previously assessed and in an additional 3 townships (Siguxi, Shangtianba, Qianwanguan) near Yuanbaoshan Township (Figure 1D). During April 7 to 15, 2020, the same 3 counties were assessed, as were 3 different townships from the previous assessment (Yongsheng, Gumi, and Jingkou) near Yuanbaoshan Township (Figure 1D). Altogether, 240 stool specimens were obtained and tested for poliovirus; all tested negative for VDPVs.

The expert panel suggested another round of environmental surveillance be conducted in 2 sites in Liangshan Prefecture. The second round of environmental surveillance was conducted during January 2020 to August 2020 in Zhaojue and Jinyang counties. Two wastewater samples were collected from each location monthly for 6 consecutive months. Twenty-four wastewater samples were collected, and no VDPVs were detected. The 2 rounds of environmental surveillance covered 13 consecutive months. In all, 4 Sabin type 1 viruses, 31 Sabin type 3 viruses, and 25 nonpolio enteroviruses were isolated. No samples were positive for type 2 poliovirus.

### Final SIA

Sichuan Province conducted a province-wide catch-up Sabin-strain IPV SIA January through May of 2021. The SIA was selective, vaccinating only children who had received fewer than 2 doses of IPV (the national schedule had been changed from one IPV dose to two in January 2020). In the SIA, 3 337 609 children had their vaccination records assessed; 1 590 151 were eligible for IPV. Among eligible children, 1 526 045 were vaccinated with Sabin-strain IPV, yielding 95.97% coverage of the target population needing one IPV dose.

### WHO Assessment

On September 6, 2020, 1 year after the investigation and response, and after 1 year of enhanced and extended environmental surveillance, the level 2 response was ended. The International Health Regulations Emergency Committee for Polio met in October 2020 and reviewed evidence from the investigation and response. Based on the evidence provided, WHO removed China from the list of countries with circulating VDPVs.

## Discussion

This polio outbreak paralyzed 1 child. China’s AFP surveillance system detected the child and triggered an enormous investigation and response that involved searching for the circulating VDPV2 in healthy children and wastewater, searching more than 30 million medical records for related cases of paralytic polio, assessing vaccination records of millions of children, vaccinating more than half a million children with a relatively new Sabin-strain IPV, conducting a year of enhanced polio and poliovirus surveillance to ensure transmission was interrupted, and ultimately supporting a change in routine polio vaccination in China to include 2 doses of IPV rather than 1 and catching up children vaccinated under the 1-IPV-dose schedule with a second dose. By using Sabin-strain IPV to successfully respond to the outbreak, deployment of a monovalent type 2 OPV in a population with low immunity to type 2 polio was avoided, which avoided the risk of generating VDPV2s associated with monovalent type 2 OPVs.

The Sichuan VDPV2 was the first VDPV2 discovered in an AFP case since the May 2016 switch from trivalent to bivalent OPV. One postswitch VDPV2 had been isolated from environmental samples in Xinjiang in April 2018 and was also responded to with an investigation and a Sabin-strain IPV vaccination campaign. After the investigation and response, no additional VDPV2s were found in Xinjiang, either in environmental samples or in AFP cases.

The source of the Sichuan circulating VDPV2 was never identified. The circulating VDPV2 was genetically related to the Xinjiang VDPV2, sharing 9 nucleotide changes from Sabin-strain type 2 OPV between the Sichuan VDPV2’s 28 nucleotide changes and the Xinjiang VDPV2’s 13 changes. However, an epidemiological link between the Sichuan and Xinjiang polioviruses was not able to be established. The 28 nucleotide changes indicate that this VDPV2 likely circulated for 3 years in Liangshan and was probably derived from trivalent OPV use, with evolution from Sabin-strain type 2 OPV to VDPV2 enabled by low routine vaccination coverage in this remote county that allowed continuous person-to-person transmission and genetic drift. That half of the young children surveyed in Liangshan had received no doses of polio vaccine supports the possibility of person-to-person transmission.

Since the switch from trivalent to bivalent OPV, many countries using bivalent OPV have experienced circulating VDPV2 outbreaks. According to the WHO, there have been 47 circulating VDPV2 outbreaks in 20 countries, with some outbreaks involving more than 1 country.^5^ The number and geographic breadth of postswitch circulating VDPV2 outbreaks exceed modeling forecasts. Most countries with circulating VDPV2 outbreaks used monovalent type 2 OPV to respond to outbreaks. However, the relative low gut immunity to type 2 poliovirus following the cessation of type 2 OPV vaccination allows type 2 OPV lineages to survive and become circulating VDPV2, potentially seeding new outbreaks.^6^ For example, detection of concurrent and independent circulating VDPV2 emergences in Angola may have been associated with monovalent type 2 OPV response–related SIAs in neighboring Democratic Republic of the Congo or related to other inadvertent Sabin-strain type 2 OPV exposures in Angola.^7^

Overall, 2 to 3 VDPV events occur every year in China, although most are not circulating VDPVs.^8^ A type 1 circulating VDPV was isolated from 2 individuals with AFP and 4 contacts in Guizhou Province in 2004, marking the first circulating VDPV detection in China. In 2011 to 2012, circulating VDPV2s (which differed from one another by 6 to 12 nucleotide changes) were isolated from 4 individuals with AFP and 1 contact between August 20, 2011, and February 8, 2012 in Aba County, Sichuan Province. Both cVDPV outbreaks were detected early and successfully responded to and interrupted with trivalent OPV campaigns.

It was a difficult choice whether to use Sabin-strain IPV or request monovalent type 2 OPV from the WHO to respond to the Sichuan outbreak. WHO Polio consultation supported using the Sabin-strain IPV since China has low risk of poliovirus transmission but cautioned that an IPV response may fail and require a subsequent monovalent type 2 OPV response. Although there are countries, such as Finland and Sweden, that eliminated poliovirus with IPV alone, evidence of sole use of IPV to successfully respond to a polio outbreak is difficult to find.^2^ Partial effectiveness of IPV in response to a 1984 outbreak in Finland was evident, but OPV was used when the outbreak spread to adults.^9^ A decision analysis for polio outbreak management in the United States after OPV was no longer licensed showed that stockpiled monovalent OPV would be the preferable vaccine for response in most scenarios, but IPV would be preferable in low-coverage scenarios.^10^

The Sichuan outbreak response used Sabin-strain IPV rather than the much more commonly used Salk-strain IPV. It is not clear whether there is an advantage of Sabin-strain vs Salk-strain IPV in a Sabin strain–derived VDPV2 outbreak. Evidence from a randomized clinical trial showed geometric mean neutralizing antibody titer of a Sabin-strain IPV–only schedule for polio type 1 and 2 were significantly higher than that of Salk-strain IPV—only schedule when antibody titers were measured with a Sabin strain–based neutralization assay.^11^ A phase 2 study indicated that compared with Salk-strain IPV, Sabin-strain IPV had similar or higher seroconversion rates and geometric mean neutralizing antibody titer against all 3 Sabin types in infants with a Sabin-strain IPV produced in the PER.C6 cell line.^12^ Practical experience is limited, however, and to our knowledge, this is the first time Sabin-strain IPV alone was used in response to a circulating VDPV2. That the Sabin-strain IPV response was successful indicates that Sabin-strain IPV may be a viable option for control of circulating VDPV2 outbreaks, at least in similar settings with low transmission potential, ability to achieve high outbreak response coverage, and with good sanitation.

To address the inherent risk of seeding new circulating VDPV2s with the use of monovalent type 2 OPV, the Global Polio Eradication Initiative fostered development of a genetically more stable monovalent type 2 OPV, called novel type 2 OPV, or nOPV2. In clinical trials, nOPV2 has been shown to be safe, immunogenic, and genetically more stable than monovalent type 2 OPV.^13^ The WHO has listed nOPV2 for emergency use in circulating VDPV2 outbreaks, and this vaccine successfully interrupted a circulating VDPV2 outbreak in Tajikistan.^14^

Our use of environmental surveillance to search for polioviruses, combined with searching in stool samples from healthy children is the most sensitive means to detect poliovirus circulation that is currently feasible. The importance of environmental surveillance in outbreak settings was reinforced in a study of the London VDPV2 investigation and response published recently in *The Lancet*.^15^ In an accompanying Comment, Drs Hill and Pollard state that the use of polio environmental surveillance in London “highlight the crucial role of environmental surveillance in identifying and understanding poliovirus transmission, and guiding appropriate and timely responses” and that “ongoing surveillance will be essential to determine if the intervention in London works or whether further escalation is needed.”^16^

### Limitations

Our study has limitations. Before the index case of paralysis, there was no environmental surveillance in Sichuan. Therefore, the duration of transmission prior to detection of the index case by the AFP surveillance system is unknown. The number of nucleotide changes indicates approximately 3 years of circulation. A related limitation is that the exact chain of transmission between Sichuan and Xinjiang could not be determined. This is likely due to the infrequency of paralysis in poliovirus infection. Furthermore, it is scientifically impossible to absolutely prove the absence of the poliovirus, implying that some postresponse poliovirus circulation may have gone undetected. However, we used the most sensitive detection methods available globally and could not detect the VDPV2. WHO Polio and WHO IHR Emergency Committee for Polio agreed with the expert panel assessment that transmission was interrupted.

## Conclusions

After apparently circulating silently for approximately 3 years following cessation of type 2 OPV in a low-coverage prefecture of Sichuan Province, a circulating VDPV2 became visible through China’s AFP surveillance system in 2019. The circulating VDPV2 was genetically linked with a then-younger VDPV2 in Xinjiang Province from the year before, and this Xinjiang VDPV2 may have originated from silent circulation in Sichuan Province. The investigation started from the index case and expanded from township to county to prefecture level, discovering 3 healthy children infected with the circulating VDPV2, but finding no evidence of the virus in wastewater. After investigation, a series of selective and nonselective SIAs, expanding from county to prefecture and accompanied by multimodal poliovirus surveillance, was used to interrupt transmission. The circulating VDPV2 has not been detected since, despite intensive search. The investigation and Sabin-strain IPV response was safe, efficient, and successful.

## Notes

Supplement 1.
Nonauthor Collaborators

Supplement 2.
Data Sharing Statement
