# Supplementary material for: Analysis of a Sabin-Strain Inactivated Poliovirus Vaccine Response to a Circulating Type 2 Vaccine-Derived Poliovirus Event in Sichuan Province, China 2019-2021
Source: JAMA Netw Open. 2023 Jan 5;6(1):e2249710. doi: 10.1001/jamanetworkopen.2022.49710 (PMC9856606; doi:10.1001/jamanetworkopen.2022.49710)
Supplement: Supplement 1. — Nonauthor Collaborators [file jamanetwopen-e2249710-s001.pdf]

Supplemental Online Content: Nonauthor Collaborators
*First name, last name, and suffix (if applicable) are required and will appear in PubMed.

***Group Name(s): China cVDPV2 response experts group**

*First Name and Middle Initial(s)	*Last Name	*Suffix (eg, Jr, III)	Academic Degrees	Institution	Location (city, state/province, country)	Role or Contribution, eg, chair, principal investigator	Group (if more than 1 Group listed in the byline) and/or Subgroup (eg, Steering Committee)
Weizhong	Yang		MD	Chinese Academy of Medical Sciences Peking Union Medical College	Beijing, China	epidemiology and immunization expert	VDPV2 group
Qun	Li		MD	Chinese Center for Disease Control and Prevention	Beijing, China	emergency response expert	VDPV2 group
Huiming	Luo		MD	Hainan Center for Disease Control and Prevention	Haikou, Hainan province, C	emergency response and polio expert	VDPV2 group
Aiqiang	Xu		MD	Shandong center for disease control and prevention	Jinan, Shandong province,	polio expert	VDPV2 group
Fang	Fang		MD, PhD	Beijing children's hospital	Beijing, China	clinical expert	VDPV2 group
