# Supplementary material for: Analysis of a Sabin-Strain Inactivated Poliovirus Vaccine Response to a Circulating Type 2 Vaccine-Derived Poliovirus Event in Sichuan Province, China 2019-2021
Source: JAMA Netw Open. 2023 Jan 5;6(1):e2249710. doi: 10.1001/jamanetworkopen.2022.49710 (PMC9856606; doi:10.1001/jamanetworkopen.2022.49710)
Supplement: Supplement 2. — Data Sharing Statement [file jamanetwopen-e2249710-s002.pdf]

# Data Sharing Statement

Yang. Analysis of a Sabin-Strain Inactivated Poliovirus Vaccine Response to a Circulating Type 2 Vaccine-Derived Poliovirus Event in Sichuan Province, China 2019-2021. *JAMA Netw Open*. Published January 05, 2023. doi:10.1001/jamanetworkopen.2022.49710

**Data**
**Data available:** No
